# Smart Livestock: A Federated Learning Based System for Real‐Time Cattle Health, Stress and Water Resource Monitoring

**DOI:** 10.1002/vms3.70979

**Published:** 2026-04-30

**Authors:** Vineeta Gulati, Rahul Grover, Naveen Kumar, Priya Jindal, Momina Shaheen

**Affiliations:** ^1^ Department of Computer Science and Engineering M. M. Engineering College, Maharishi Markandeshwar (Deemed to be University) Mullana Ambala Haryana India; ^2^ Department of Civil Engineering M M Engineering College, Maharishi Markandeshwar (Deemed to be University) Mullana Ambala Haryana India; ^3^ Chitkara University Institute of Engineering and Technology, Chitkara University Punjab India; ^4^ Chitkara Business School Chitkara University Punjab India; ^5^ School of Arts, Humanities, and Social Sciences University of Roehampton London UK

**Keywords:** artificial intelligence, federated learning, internet of things, machine learning, smart agriculture

## Abstract

**Background:**

Centralized machine learning (ML) models often face challenges related to data privacy, messaging latency and limited internet access in rural areas. To address these issues, a federated learning (FL) based architecture for real‐time detection of health and stress in cattle using sensors is deployed. This model enables edge devices such as smart collars, wearable sensors and cameras, to collaboratively learn a global model without sharing raw data.

**Objectives:**

To create a FL‐enabled architecture of real‐time health and stress detection in cattle with distributed smart farming devices, and to deal with the issue of data privacy, latency and poor internet in rural conditions.

**Methodology:**

In the proposed system, FL is used to allow edge devices, including smart collars, wearable sensors and cameras to jointly train a global model without exchange of raw data. There is the utilization of multimodal time‐series data, such as temperature, heart rate, motion trajectories and environmental conditions. The architecture combines LSTM and CNNs to find anomaly and behaviour patterns. Measurement of performance is compared with centralized ML models with real or simulated livestock data.

**Results:**

The results of an experiment prove that the FL‐based model has better performance than centralized and baseline federated models and this has an accuracy of 93.1%. The model demonstrates better convergence properties and saves a lot in the transmission of the raw data. It is applicable in cattle, early stress detection, illness and abnormal behaviour.

**Conclusion:**

The possibility of the use of federated AI systems provides an accurate solution to secure, privacy‐preserving and efficient livestock monitoring. The suggested solution can contribute to the improved economic performance and animal welfare in the future since it promotes sustainable smart agriculture and can be integrated with systems like managing irrigation in the future.

## Graphical abstract

This study introduces a federated learning‐based system for real‐time cattle health and stress detection using smart farming sensors. It preserves data privacy and reduces latency by training models on edge devices. Combining LSTM and CNN, it achieves 93.1% accuracy, enabling early illness detection and promoting sustainable livestock management.

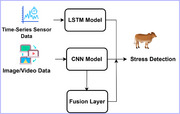

## 1 Introduction

Smart livestock farming, a revolutionary technology in agriculture, applies intelligent tools for real‐time health and stress monitoring in cattle using FL. This ML solution enables the collaboration of distributed sources of farm data to construct prediction models and secure privacy and safety of the data. Using a combination of IoT devices, ML algorithms and FL methods, farmers are able to monitor the health and welfare of their animals and improve the productivity and well‐being of animals (Arshad et al. 2024; Bernabucci et al. 2025; García et al. 2020).

**FIGURE 1:**
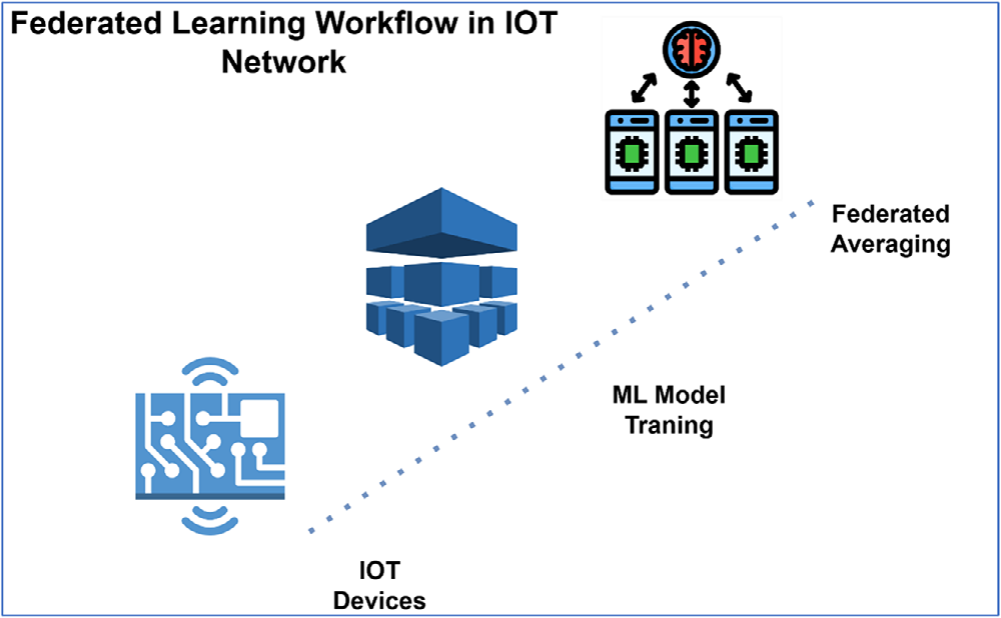
FL and IoT edge devices enabled an integrated framework for distributed model training.

The increasing popularity of smart livestock farming can be explained by the fact it reshapes the conventional mode of farming by offering real‐time health condition of cattle due to its physiological and behavioural indicators. The technology allows early disease diagnoses and prevention of stress which are essential in the welfare and yield of the farm (de Oliveira et al. 2024; Linstadt et al. 2024; Dzermeikaitė et al. 2023). These monitoring systems have the ability to learn a great deal of data to reveal patterns that point to health‐related issues, and with the help of AI and ML, farmers can make informed decisions based on data (Linstadt et al. 2024). Figure 1 illustrates the FL architecture that has IoT edge devices to train distributed models.

### 1.1 Federated Learning

**FIGURE 2:**
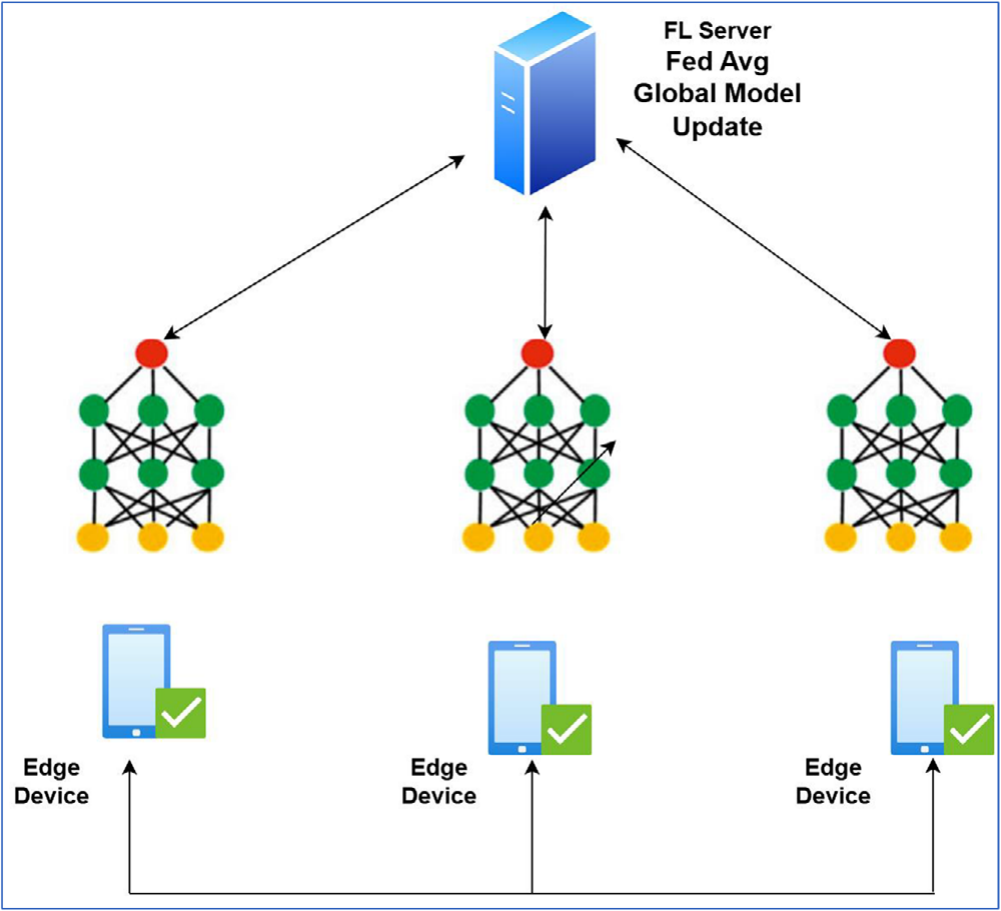
Operational workflow of the FL server with the FedAvg aggregation method.

It is an emerging ML paradigm that enables collaborative model training among multiple decentralized devices while preserving privacy and data security (Zhang et al. 2021). This approach allows training algorithms to use local data samples available at individual devices or servers, rather than storing data on a central server as shown in Figure 2. The advantage of FL lies in its ability to handle data heterogeneity, as data from different clients may not be identically and independently distributed, resulting in models with strong generalization capabilities (D. C. Nguyen et al. 2022).

In FL, clients train local models on their own data and send model updates, rather than raw data, to a central server. Secure aggregation of these model parameters produces a global model that leverages information from all clients. This learning by doing system enables continuous improvement of model performance while maintaining client data confidentiality (Antunes et al. 2022).

### 1.2 Role of IoT in Smart Livestock

Smart farming is enabled through the IoT, where it collects diverse real‐time data by means of wearables, sensors and edge devices. In cattle farming, wearables are used to monitor physiological parameters such as heart rate, body temperature and motion profiles. Temperature information, flowmeters, soil moisture sensors allow efficient distribution of water in irrigation systems to maximize crop productivity and to conserve resources.

Sophisticated processing is required to extract meaningful information from the high‐frequency, multimodal data produced by these systems. IoT networks associated with FL allow for distributed learning where edge nodes collaborate to train models without sharing raw data, preserving privacy and reducing communication overheads.

### 1.3 Research Gap

Smart livestock farming management systems have shown great potential in real‐time farm operations decision support, early disease prediction using behavioural and physiological cues, detection of livestock health and stress using multimodal data, and efficient scheduling of irrigation using soil and weather analytics. Despite these advancements, several issues still remain to be resolved:

- Monitoring of irrigation and livestock are not fully integrated into a single federated system.
- Difficulties managing non‐IID data across several farm devices.
- Absence of low‐latency, real‐time communication protocols that guarantee energy efficiency and scalability.
- There is limited empirical research in a privacy‐preserving FL environment that has compared the spatial feature CNN to the time series LSTM.

By creating a unified FL‐based system that can monitor water supplies and cow health in real‐time while protecting privacy, this research seeks to close these gaps.

### 1.4 Research Contribution

The key findings in this work are as follows:

- An architecture proposing privacy‐preserving federated learning to smart livestock monitoring is discussed that allows training a distributed model with several farms without transferring the raw sensor data.
- A hybrid model of deep learning that has been built that incorporates LSTM and CNN deep learning models is meant to be able to reflect both the temporal physiological trends and possibly spatial behavioural information based on multimodal livestock data.
- The federated CNN–LSTM framework is tested on the MmCows multimodal data in simulated multi‐farm federated state and shows better results (93.1% accuracy and 0.91 F1‐score) when compared to the centralized and conventional machine learning models.

The suggested system proposes a scalable smart livestock monitoring system that can work in the rural setting with low connectivity but maintain the privacy of the farm‐level information. In contrast to the traditional centralized livestock monitoring systems, the paper examines a federated learning system of multimodal cattle health monitoring in distributed farms. The given strategy will allow training the models in cooperation and keeping the local farm data confidential, an issue that has become a major hindrance in the widespread implementations of smart livestock.

## 2 Related Work

AI and IoT combined with privacy preserving ML has brought forth a promising development for precision livestock farming. In this section, authors first examine the existing works on livestock monitoring in IoT era, then provide an overview of traditional machine learning algorithms, in addition, authors review some deep learning techniques in health and stress detection. Table 1 represent the summary of related work in smart livestock monitoring and Table 2 represent its contextual comparison.

**TABLE 1:** Correlation with representative related work in smart livestock monitoring.

Category	Study	Technique/model	Application/contribution	Limitations
IoT‐based applications in livestock monitoring	Simanungkalit et al. (2021)	Wearable sensor network	Real‐time cattle body temperature and movement monitoring	Centralized data storage; scalability and privacy issues
	Farooq et al. (2022)	RFID + acceleration data	Classification of livestock activities	No privacy protection; relies on central server
	Unold et al. (2020)	Review study	IoT architectures, protocols and applications in cattle monitoring	Theoretical review; lacks implementation
	Post et al. (2020)	CowMonitor (wearable + cloud)	Detect oestrus and mastitis using IoT and cloud‐based decision trees	Centralized; energy‐ and data‐intensive
ML approaches for animal health detection	Shahinfar et al. (2021)	SVM	Detection of lameness in dairy cows via locomotion score	Needs central data aggregation
	Guitian et al. (2023)	ML on images and sensor data	Disease detection in cattle	Poor edge applicability; dependent on infrastructure
	Bensakhria (2023)	Decision trees, SVM, clustering	Surveillance, diagnosis and outbreak prediction	Requires expert integration; lacks decentralization
Deep learning for stress and behaviour analysis	Wu et al. (2022)	CNN	Posture classification using cattle images	High computation; centralized, labelled data needed
	Acikmese and Alptekin (2019)	LSTM	Monitoring cattle stress using physiological time‐series data	Centralized model training; no privacy
	Khan et al. (2022)	CNN, LSTM, CNN–LSTM	Stress detection using mobile sensor data (human study)	Indirect livestock relevance; privacy concerns
FL in smart agriculture and animal husbandry	Mao et al. (2022)	FL	Crop disease detection via drone images	Not livestock‐focused
	Arshad et al. (2023)	FL + accelerometers	Intelligent poultry farm monitoring	Domain‐specific; not multimodal
	Dembani et al. (2025)	FL + body sensors	Cattle health monitoring with FL	No multimodal data fusion or stress detection
	Praharaj et al. (2025)	Survey	FL in smart farming; privacy‐preserving model training	Survey; lacks empirical deployment
	Tangorra et al. (2024)	FL + transfer learning and compression	Efficient multi‐farm training with reduced comm. overhead	No specific stress monitoring implementation

*Note*: The listed studies use various datasets, sensing modalities and evaluation metrics; thus, comparison is done on the contextual positioning as opposed to actual performance benchmarking.

**TABLE 2:** Contextual comparison of related research on smart livestock monitoring.

Ref.	Year	Technology/model used	Sensors/devices	Data type	Implementation	Performance evaluation
Ramli et al. (2020)	2018	Wearable sensor network	Body sensor	Temp., motion	✓	✗
Simanungkalit et al. (2021)	2019	RFID + accelerometer	RFID tags	Motion	✓	✗
Farooq et al. (2022)	2019	IoT Arch. review	Various	Multi	✗	✗
Unold et al. (2020)	2020	CowMonitor (BLE + cloud)	Wearable + BLE	Behaviour, health	✓	✓
Post et al. (2020)	2018	SVM (ML)	Sensors	Movement	✓	✓
Shahinfar et al. (2021)	2019	ML on image/sensor data	Cameras, sensors	Images, signals	✓	✓
Guitian et al. (2023)	2020	SVM, decision trees, clustering	Digital health records	EHR, logs	✓	✓
Bensakhria (2023)	2020	CNN (DL)	Image sensors	Images	✓	✓
Wu et al. (2022)	2021	LSTM (DL)	Bio‐sensors	Time‐series	✓	✓
Acikmese and Alptekin (2019)	2021	CNN–LSTM (DL)	Passive mobile sensors	Mobile data	✓	✓
Khan et al. (2022)	2021	FL	Drone imagery	Images	✓	✓
Mao et al. (2022)	2022	FL + accelerometers	Wearables	Motion	✓	✓
Arshad et al. (2023)	2022	FL + body sensors	Body area networks	Bio‐signals	✓	✓
Dembani et al. (2025)	2023	Survey on FL in agri	Multiple	Multi	✗	✗
Praharaj et al. (2025)	2023	FL + transfer learning	Multi‐farm	Multi	✓	✓
Our work	2025	FL + LSTM‐CNN	Smart collars, wearable sensors, env. sensors	Physiological, environmental, video	✓	✓ (93.1% accuracy, 0.91 F1)

*Note*: The listed studies use various datasets, sensing modalities and evaluation metrics; thus, comparison is done on the contextual positioning as opposed to actual performance benchmarking.

### 2.1 IoT‐based Applications in Livestock Monitoring

The monitoring of livestock behaviour and physiological states via IoT technology is prevalent. For example, a wearable sensor network for real‐time cattle body temperature and movement measurement is proposed in (Ramli et al. 2020). RFID and acceleration data to class livestock activities is used in (Simanungkalit et al. 2021), but most of these systems use centralized storage, which brings with its scalability and privacy issues.

The utilization of IoT technology in animal production and precision livestock farming, concerning follow and health condition tracking as well as environmental conditions, is becoming ubiquitous. A review of IoT reference architectures, communications protocols and devices and their applications in real‐time cattle monitoring is proposed in (Farooq et al. 2022). Similarly, an entire IoT‐based cow health monitoring system, ‘Cow Monitor’, incorporating wearable inertial sensors (cow devices), BLE‐enabled hubs, cloud infrastructure, as well as an android app for online behaviour and oestrus validation is proposed in (Unold et al. 2020). This system showed a good performance in identifying health problems (e.g., oestrus and mastitis) with onboard processing and cloud‐based decision trees. However, these solutions are not without limitations, as they are often dependent on a centralized cloud server for storing and analysing sensory data issues poised by the very practical concerns of data privacy, energy efficiency, safety and real‐time analytics in resource‐constrained settings such as remote farms, emphasizing the importance of decentralized, privacy‐preserving alternatives, such as FL.

### 2.2 ML Approach on Animal Detection of Health

ML methods including SVM (support vector machine), decision trees and k‐NN (k‐nearest neighbors) have been used for the detection of livestock health status and behaviour prediction. For instance, SVM to detect lameness in dairy cows based on locomotion score is used in (Post et al. 2020). ML algorithms for disease detection in cattle using images and sensor data is applied in (Shahinfar et al. 2021). Even though these models work well, they need a centre for data aggregation and they cannot be used in peripheral region when infrastructure is poor.

Machine learning in animal and veterinary public health surveillance, with diagnosis, outbreak prediction and health risk assessment the principle uses is reviewed in (Guitian et al. 2023). Their study showed how supervised and unsupervised ML methods (e.g., decision trees, support vector machines, clustering algorithms) have been able to assist in disease prediction, syndrome detection and complex data mining, for example, from abattoir records (ARs) or electronic health records (EHRs). They also emphasized the need to combine ML with domain expertise for successful disease surveillance and control measures at both the farm and policy levels.

### 2.3 Deep Learning for Stress and Behavioural Analysis

Deep learning models generalize better on complex livestock data. CNN for categorizing cattle posture from image data sets is used in (Bensakhria 2023) and an LSTM to the task of monitoring the stress in dairy cattle from the time‐series of physiological data is applied in (Wu et al. 2022). High performance of such models, however, comes at a cost as they also require considerably computational resources and are based on labelled data and centralized training, which in turn adds to privacy issues and low efficiency.

Physiological stress recognition with passive mobile sensor data has also been tested on deep learning models of LSTM, CNN and CNN‐LM configurations (Acikmese and Alptekin 2019). The experiment showed that LSTM was useful in modelling time‐varying parameters in multimodal sensor stream activity and audio records. These results suggest that sequence‐based models may be used to model behaviour and physiological stress patterns of real‐world sensor data, which makes them applicable to the analysis of stress in livestock.

### 2.4 FL in Smart Agriculture and Animal Husbandry's Scenario and Background

FL is capable of training on a joint model collaboratively without exchanging raw data, thereby maintaining privacy. An FL model for the purpose of crop disease detection from drone images is established in (Khan et al. 2022). FL to intelligent poultry farms through distributed accelerometer data is extended in (Mao et al. 2022). FL for monitoring cattle health with body area sensors is presented in (Arshad et al. 2023). However, the use of FL in multimodal cattle stress monitoring framework using deep learning is still under‐investigated.

The recent use of FL in agriculture support multi‐farm model training in a nature that optimizes data confidentiality (Dembani et al. 2025). FL is dedicated to addressing critical issues in smart farming and animal husbandry where sensitive data (e.g., soil conditions, animal health, yield estimates) can be kept local but still contribute to global models. Use‐cases range from crop yield estimation, detection of pests and diseases, to precision livestock management. However, challenges exist, such as the heterogeneity of the data, poor connectivity in rural regions and the requirement for scalable, privacy‐preserving architectures that are agriculture‐specific.

FL has become an important technology in privacy‐preserving collaborative intelligence for smart agriculture and animal husbandry. With this solution, multiple farms can train a model locally using highly sensitive sensor data, such as metrics around the health of livestock or the condition of a crop, without having to hand over the raw data. Recently, researchers have utilized FL in CSF scenario, where small farms share their data in under to build intelligent models while protecting themselves from the cybersecurity threats and reducing the communication (Dembani et al. 2025; Praharaj et al. 2025). The combination of transfer learning and model compression in these systems also improves performance with minimized local training time to enable deployability in scale to resource‐restricted farm environments.

## 3 Proposed System Architecture

This research proposes a comprehensive model integrating IoT, ML and FL for health and stress monitoring in smart livestock farming. The system supports real‐time, privacy‐preserving data processing on decentralized edge devices such as smart collars, wearable sensors and environmental monitoring devices. The method consists of five key steps, namely the collection of data with the help of IoT devices, the training of local ML models, federated aggregation, model convergence through iteration and the deployment of the system.

**FIGURE 3:**
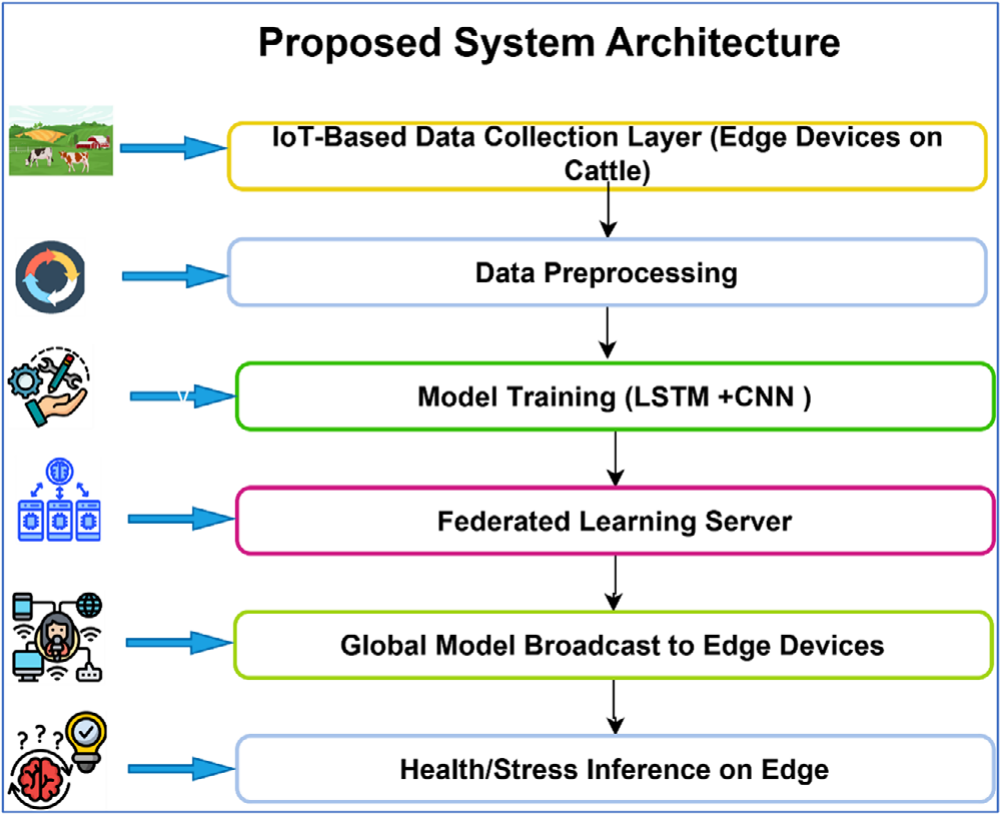
The generalized structure of the proposed FL‐IoT enabled smart livestock monitoring system.

In Figure 3, the generalized architecture of the proposed smart livestock monitoring system using FL has been illustrated. The suggested architecture takes multiple sensor streams in the form of multimodal data and processes them with a temporal deep learning model. The LSTM layer obtains time‐related patterns of behavioural and physiological dynamics, whereas the federated learning model allows distributed training of farm clients. Model updates are done on a per‐client basis, and they are displayed infrequently to a central server. The system provides privacy of the data by ensuring that the data of animals and farms are localized to maintain privacy of the data, and to prevent communication overhead. This allows the system to be scaled and implemented in large farms. Lightweight models, optimized training loops, and low‐power edge device compatibility enable practical usage and sustainable applications in real‐life scenarios.

### 3.1 Data Acquisition of IoT in Livestock Scenarios

Wearable and sensory IoT modules are attached to individual cattle and farm equipment to collect multimodal time‐series data in real‐time (Tangorra et al. 2024). Physiological parameters, behavioural data and ambient conditions are sensed and logged. Each device maintains local data: $X_{y}$.

### 3.2 Training ML Models on Edge Devices

Each edge device or gateway node is equipped with limited computing power to perform local training on collected data. This study employs a hybrid deep learning model, CNN–LSTM. CNNs capture spatial features from camera or sensor data, while LSTM layers model temporal dependencies in behaviour over time. This study uses LSTM networks to predict time‐series IoT sensor data in terms of heart rate, body temperature and activity levels that vary across time. These physiological parameters tend to vary gradually and not suddenly and such early signs of stress or disease progression may only be realized when historical trends are taken into consideration. The LSTM model facilitates proper health monitoring by allowing the temporal dependencies to be observed between successive sensor readings, which consequently allows detecting slow physiological changes that could be manifested in either stress or malaise in cattle.

Each device trains its own model independently, as the monitored environmental and physiological activities of the cattle differ (Gao et al. 2023). For each Device *y*, the model is trained to minimize the local loss function as shown in Equation (1) with input vector (*w*_i_), class label (*z*_i_), Parameter (*k*) and chosen loss function (*q*). In Equation (2) $Z^{\Lambda }$ is the predicted probability vector for health conditions. Table 3 represents the notations used in Equations (1) and (2).

(1) $$L_{y}\left(k\right)=\frac{1}{\left|X_{y}\right|}\sum q\left(w_{i},k\right),z_{i}$$

(2) $$Z^{\Lambda }=\mathrm{Softtmax}\left(k\cdot \mathrm{LSTM}\left(\mathrm{CNN}\left(w\right)\right)+b\right)$$

**TABLE 3:** Notations used in Equations (1) and (2).

Variable	Description
*L*_y_	Local loss function computed on Device *y*
*w*_i_	Input feature vector for the *i*th data sample
*z*_i_	True class label associated with input *w*_i_
*K*	Number of data samples on the local device
*Q*	Chosen loss function (e.g., cross‐entropy, MSE)
*F*(wi)	Output of the model given input *w*_i_​
Z^	Predicted probability vector for health conditions
Softmax(.)	Activation function to convert logits into class probabilities

### 3.3 Federated Learning and Model Averaging

To protect data privacy and address network conditions typical of rural farming areas, the proposed framework adopts FL. Instead of sending raw sensor data, each device securely transmits model updates to a central aggregation server using encrypted protocols. After local training, Device y shares its updated parameters $k_{y}^{\left(t\right)}$ at Round *t* and calculate the federated averaging as shown in Equation (3). Table 4 represents the notations used in Equation (3).

**TABLE 4:** Notations used in Equation (3).

Variable	Description
*k*_y_^t^	Local model parameters of Device *y* at Round *t*
*k* ^t^	Global model parameters after aggregation at Round *t*
*X*_y_ or *X*j	Number of local data samples on Device *j* or *y*
FedAvg	Federated averaging algorithm for global parameter update

*Federated averaging (FedAvg)*:

(3) $$k^{\left(t+1\right)}=\sum_{y=1}^{y}\frac{\left|X_{y}\right|}{\sum_{i=1}^{y}\left|X_{j}\right|}k_{y}^{\left(t\right)}$$

**ALGORITHM 1:** Federated learning training procedure.

**Input**: Global model parameters k0 Number of communication rounds T Local training epochs E Number of clients Y **Output**: Final global model kT *Initialize global model parameters k0* *Repeat for each communication round t = 1 to T do*: *Server selects a subset of available clients* *For each client y in parallel*: *Receive global model k_t_* *Perform local training of E epochs on the client's local dataset* *Send update to server about parameters Ky^t* *Server aggregates client models using FedAvg*: $k(t+1)=\sum \left(|\mathrm{Xy}|/\sum \left|\mathrm{Xj}\right|\right){\mathrm{ky}}^{t}$ *Return final model KT*

The server aggregates all updates using the federated averaging (FedAvg) algorithm to obtain the updated global model (Algorithim 1). This global model is then transmitted back to the devices, triggering the next round of training. This process ensures that sensitive farm data remains decentralized and mitigates latency and bandwidth constraints.

### 3.4 Iterative Federated Optimization and Convergence

An iterative algorithm is used to solve the minimization problem to arrive at the convergence. Federated training is done through multiple communication rounds. During a round, devices train the local model in a given number of epochs and update them on the server. Critical hyperparameters (learning rate, batch size and local epochs) are optimized so that fast convergence is achieved, even in the case of low connectivity. The training process will be repeated until the accuracy or loss reaches a common value, or the only weak improvements are registered.

This approach provides a privacy‐preserving, decentralized and intelligent solution for cattle health and stress monitoring. By utilizing IoT for real‐time data acquisition, ML for behaviour analysis and FL for joint model training without data exposure, the proposed framework addresses key challenges in smart livestock farming: data privacy, communication efficiency and model generalization across diverse farm settings.

## 4 Dataset and Preprocessing

### 4.1 Dataset Description

To assess the proposed federated learning based smart livestock health monitoring system, authors employed the MmCows (multimodal cattle) dataset, which is a multimodal dataset that combines physiological, behavioural and environmental data gathered through wearable IoT devices deployed on dairy cattle. The data consists of time‐series sensor data, which replicate actual agricultural environments and can be used in early stress and disease detection (Vu et al. 2024). The data set consists of a set of diverse characteristics that define the health, activity and environment of an individual animal. Upon the MmCows dataset, there is no clear ground‐truth annotation related to cattle stress. Thus the stress tags in this research were obtained based on proxy physiological reactions that are usually linked to stress reaction in cattle including an increase in heart rate (> 100 bpm) and an increase in body temperature (> 39.5°C) as per veterinary guidelines reported in previous research (Polsky and Von Keyserlingk 2017l; Ji et al. 2020). These labels are to be understood as the signs of possible physiological stress but not clinical diagnoses. Table 5 lists the main parameters that we made use of in our work.

**TABLE 5:** Features description of the dataset along with their code.

S. no.	Feature name	Feature code	Definition
1	Timestamp	timestamp	Date and time at which the sensor reading was recorded
2	Cattle ID	cattle_id	Unique identifier assigned to each cow in the dataset
3	Heart rate	heart_rate	Cow's heartbeats per minute (bpm); higher than normal indicates stress
4	Body temperature	body_temp	Internal body temperature in °C; fever may indicate illness
5	Activity level	activity_level	Numeric representation of movement level from accelerometer data
6	Ambient temperature	ambient_temp	Environmental temperature in °C
7	Humidity	humidity	Relative humidity in percentage (%)
8	Latitude	location_lat	GPS latitude coordinate of the cow's position
9	Longitude	location_long	GPS longitude coordinate of the cow's position
10	Stress indicator	is_stressed	Proxy stress label 1 = physiologically stressed, 0 = normal

The data was taken from various IoT devices used in smart farming context. Such devices allow for real‐time receipt of multimodal data which are essential for timely health assessment. Table 6 shows the types of components related to IoT, and the role of each on the system.

**TABLE 6:** Types of IoT components with corresponding sensor device and functionality.

S. no.	IoT component	Sensor type/device	Purpose/functionality	Data generated
1	Wearable smart collar	Heart rate sensor, Temp sensor	Monitors vital signs like heart rate and body temperature to detect stress or illness	heart_rate, body_temp
2	Accelerometer unit	3‐Axis accelerometer	Detects movement patterns to identify abnormal activity or inactivity	activity_level
3	GPS tracker	GPS module (GNSS)	Tracks cattle movement and location for grazing monitoring and stress‐related behaviour	location_lat, location_long
4	Environmental monitor	Temp and humidity sensors	Monitors environmental stressors (e.g., heat, humidity)	ambient_temp, humidity
5	IoT gateway device	Edge‐computing‐enabled device	Aggregates sensor data, enables FL and supports privacy‐preserving analytics	Aggregated sensor data
6	Camera module	CCTV or mobile cameras	Captures behavioural video data for posture and activity detection	Video frames or derived behaviour
7	Edge/cloud storage	Local/cloud‐based server	Stores preprocessed data and trains local models for FL	Model weights, metrics

### 4.2 Data Preprocessing

A standard preprocessing pipeline was implemented to ensure the quality and reliability of time‐series sensor data used for cattle stress detection. Initially, when missing values occurred due to transmission lags or sensor dropouts, forward and backward filling strategies were applied to maintain temporal continuity without artificial interpolation. Next, outliers were removed using *z*‐score thresholding (e.g., for heart rate values), excluding physiologically implausible data (> 3 standard deviations) likely caused by sensor failure.

The cleaned data underwent min–max normalization, standardizing all features (heart rate, body temperature and activity level) to a [0, 1] interval. This step ensured numerical stability and enhanced the convergence speed of learning models. To improve model accuracy and incorporate characteristic temporal behaviour, feature engineering was applied, including rolling mean and activity variance calculations over 30‐min windows.

Finally, a binary stress label was created by thresholding related physiological criteria (e.g., heart rate > 100 bpm or temperature > 39.5°C) based on veterinary expertise, enabling the training of supervised classifiers. This preprocessing architecture maintains temporal consistency and data quality across all edge devices within the federated learning system. Table 7 shows the features transformations before and after preprocessing.

**TABLE 7:** Feature transformations before and after preprocessing.

S. no.	Step	Heart rate (bpm)	Temperature (°C)	Activity level	Note
1	Raw data	72	38.5	Low (0.2)	Missing in next row
2	Missing value treatment	NaN → 72	38.7	NaN → 0.2	Forward filled
3	Outlier handling	175 → dropped	38.9	0.4	Outlier removed (*Z* > 3)
4	Normalization	72 → 0.48	38.6 → 0.42	0.2 → 0.25	Min–max scaled (0–1)
5	Feature engineering	—	—	—	Rolling mean/variance added
6	Label generation	—	—	—	is_stressed = 1

**FIGURE 4:**
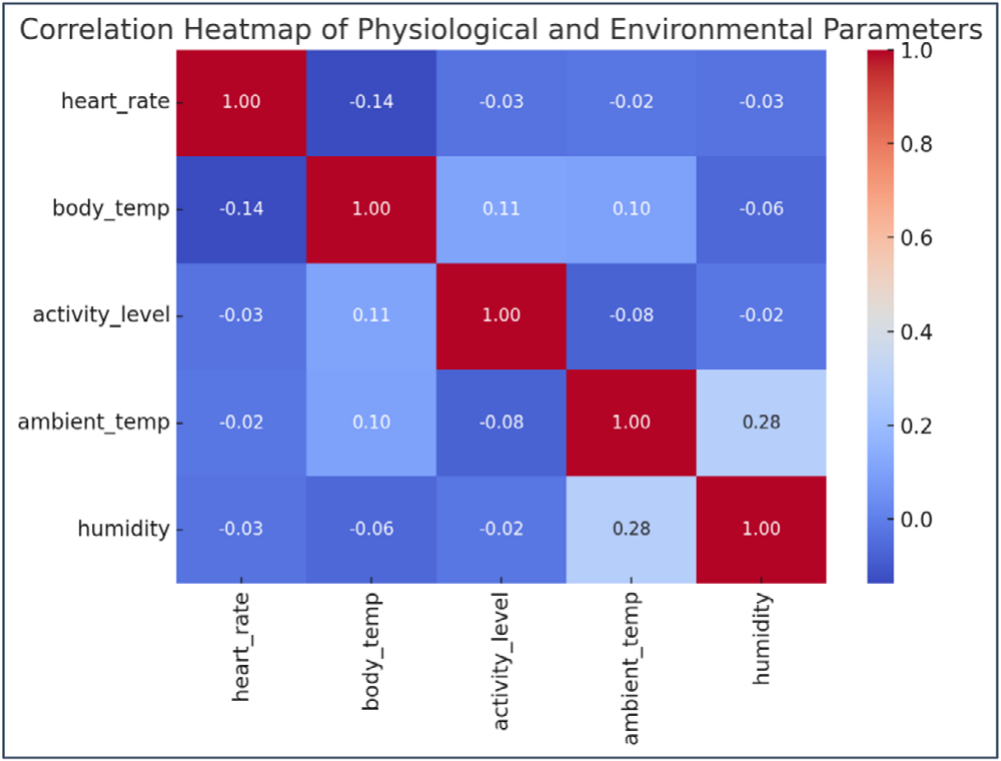
Correlation heatmap of features.

The findings from exploratory data analysis (EDA) contribute to corroborate the significance of physiological and ambient parameters and to motivate the choice of features in the subsequent training process of a prediction model. Authors computed bivariate correlations using Pearson correlation coefficients between variables including heart rate, core body temperature, activity, ambient temperature and humidity. Results were plotted by heatmap for illustration as shown in Figure 4.

## 5 Machine Learning Models

**FIGURE 5:**
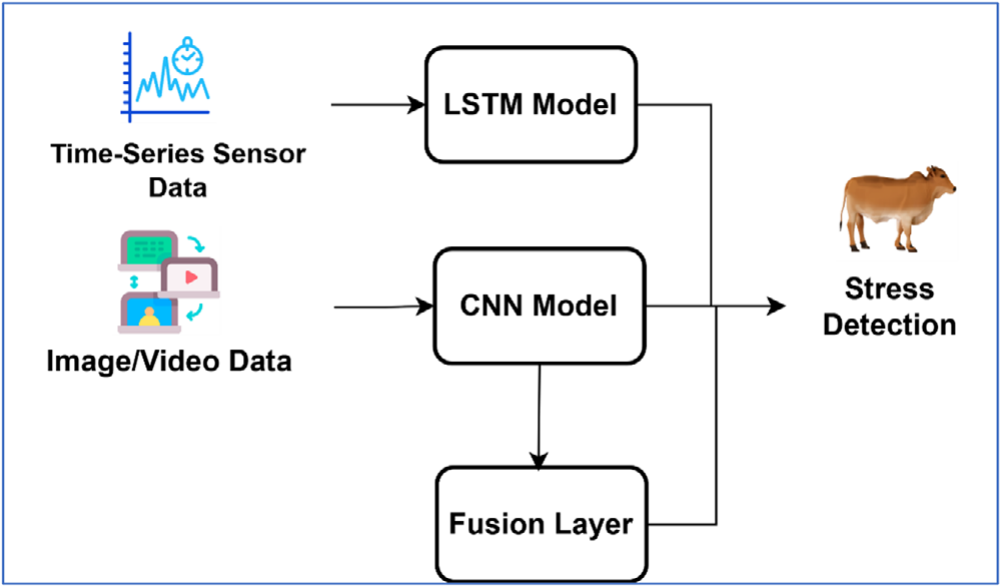
Extensible CNN–LSTM model framework.

As smart livestock monitoring devices can offer multimodal data such as heart rate, body temperature and motion pattern, The proposed structure will help to facilitate multimodal learning with the LSTM‐processing of time‐series sensor data and a visual data option with a CNN module. In the present experiment assessment of the MmCows dataset, sensor based time‐series inputs are used only because there are no raw image or video streams. we use a hybrid ML method by combining LSTM with CNN to accomplish effective processing on the multimodal data as shown in Figure 5. The models are trained in a FL system, where FedAvg aggregation is used in FL to maintain data privacy and to allow the decentralized learning at the edge devices.

LSTM has its gated memory cell structure that selectively remembers or forgets information with the passage of time. Only three main gates constitute an LSTM cell, namely, the input gate, the forget gate and the output gate, as these gates control the information flowing in, out of and within the cell state. This gating scheme allows LSTM networks to maintain past trends at long sequences, and therefore, it is very effective when it comes to time‐series data analysis.

LSTM models are appropriate for timeseries data representing IoT sensor readings (e.g., heart rate, temperature and activity levels). They still preserve temporal dependencies which is important for sensing slow physiological modifications that might indicate stress or malaise (H. H. Nguyen and Vo 2024). LSTM cell perform following operations as represented in Equations ((4), (5), (6)) with Input ($x_{t\;}$), hidden ($h_{t\;}$), cell state ($c_{t\;}$) at Time *t*, described in Table 8.

(4) $$\mathrm{Input}\;\mathrm{gate}\;:i_{t}=\sigma \left(w_{i}.\left[h_{t-1},x_{t}\right]+b_{i}\right)$$

(5) $$\mathrm{Output}\;\mathrm{gate}\;:o_{t}=\sigma \left(w_{o}\;.\;\left[h_{t-1}\;,x_{t}\right]+b_{o}\right)$$

(6) $$\mathrm{Cell}\;\mathrm{state}\;\mathrm{update}:C_{t}=f_{t}\times C_{t-1}+i_{t}\times \approx C_{t}$$

**TABLE 8:** Notations used in Equations ((4), (5), (6)).

Variable	Description
*x*_t_	Input vector at Time Step *t*
*h*_t‐1_	Hidden state from previous time step
*C*_t‐1_	Cell state from previous time step
*f*_t_	Forget gate output at Time *t*
*i*_t_	Input gate output at Time *t*
*o*_t_	Output gate output at Time *t*
*C*_t_	Updated cell state
*σ*	Sigmoid activation function

If there is any image or video data (e.g., posture, facial expression, cattle gait), we will make use of CNN to capture the spatial representation of stress or sickness through the taught features and operations include kernel, Input *z* and Feature *y* as shown in Equation (7) and description in Table 9.

(7) $$y=\;\mathrm{ReLU}\left(w\times z+b\right)$$

**TABLE 9:** Notations used in Equation (7).

Variable	Description
*z*	Input image or frame (e.g., posture, face, cattle gait)
*y*	Feature map generated after convolution
*	Convolution operation
CNN role	Extracts spatial representations (edges, patterns) from image data

This infrastructure permits a complete cattle stress and health analysis. Every model is locally trained on edge devices, their parameters are then averaged and centralized using the federated averaging (FedAvg) technique with the aim of learning a global and resilient model to protect data privacy. Table 10 shows the model comparison along with data type and their role on device.

**TABLE 10:** Model comparison by data type, functional role and deployment device.

Model	Data type	Role	Device
LSTM	Time‐series (HR, Temp, activity)	Detect physiological stress patterns	On‐device (edge)
CNN	Optional images/video	Detect visual stress indicators	On‐device (edge)
Fusion	LSTM + CNN	Unified stress detection	On‐device (edge)
FedAvg	Model weights	Global aggregation	Central server

## 6 Results and Discussion

A series of controlled experiments were carried out to validate the efficiency of the federated learning framework suggested and using the MmCows dataset which has several features, including heart rate, body temperature, the degree of activities, the temperature and humidity in the environment. Such records were on livestock wireless sensors during a given period of observation. The authors also preprocessed the data thoroughly in order to achieve reliability.

Experimental environment was an emulated distributed smart farming environment that featured a number of edge devices that served as federated clients. There are 10 federated clients in the scope of which a separate farm node with locally partitioned sensor data is represented. The Adam optimizer and binary cross‐entropy loss function were used to train the models over 50 communication rounds, each of which consisted of five local training epochs. We developed and tested three models: a centralized machine learning model, an LSTM federated learning model and an LSTM‐CNN hybrid FL model. The LSTM network modelled temporal characteristics in sensor readings, while the CNN component simulated video surveillance as additional information. The federated models, trained locally on simulated edge nodes, were aggregated using the FedAvg algorithm.

The federated LSTM‐based model, which is developed within the CNN–LSTM extensible architecture, outperforms LSTM‐only federated model and centralized model, with 93.1% accuracy and F1‐score of 0.91, as depicted in Table 11. The LSTM‐only FL model attained 89.5% accuracy and a 0.86 F1‐score. While the federated models experienced slightly increased latency (120–135 ms) and moderate bandwidth usage (7.5–8.4 MB), these operational constraints remain acceptable for rural deployments, considering the enhanced data privacy.

**TABLE 11:** Comparative performance of centralized and federated learning models with LSTM and CNN.

Model	Accuracy (%)	F1‐score	Average latency (ms)	Bandwidth usage (MB)
Centralized ML	91.2	0.88	—	—
FL (LSTM only)	89.5	0.86	120	7.5
**FL (LSTM‐based, CNN‐extensible)**	**93.1**	**0.91**	**135**	**8.4**

The bold values show that the FL (LSTM‐based, CNN‐extensible) model is the best, with the highest accuracy and F1‐scores. This demonstrates a better prediction accuracy and the slight increase in latency and bandwidth is justified by the trade‐off.

The experimental findings indicate that the federated LSTM‐based model is useful in modelling temporal patterns in multimodal livestock sensor data. The attained 93.1% accuracy implies that the predictive performance can be preserved without losing farm‐level data privacy through the decentralized training.

**FIGURE 6:**
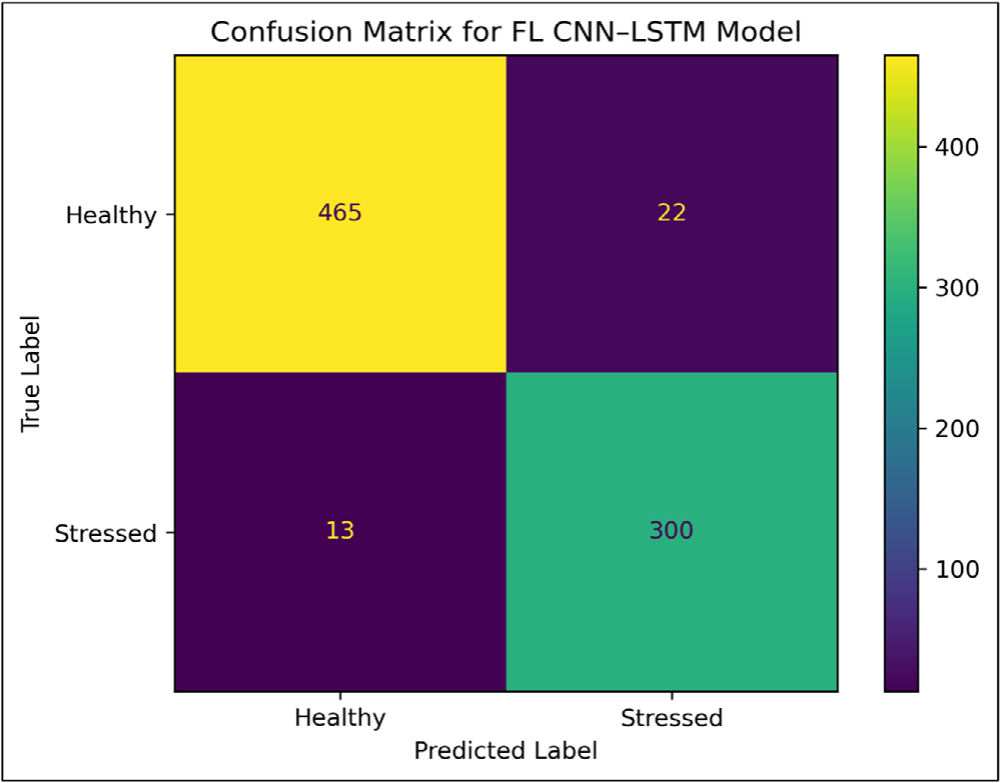
Confusion matrix for the proposed federated CNN–LSTM model employed in cattle health classification.

Figure 6 presents the confusion matrix for the proposed Federated LSTM model employed in cattle health classification. The model classified most of the instances for healthy and stressed cattle, which shows that misclassification occurred only on a few test samples. The given model's high true positive and true negative rates prove its efficiency in terms of distinguishing the stress‐related physiological and behavioural patterns. Especially significant is the low rate of false negatives, which reduces the possibility of missing early symptoms of stress or disease when monitoring the health status of the livestock.

**FIGURE 7:**
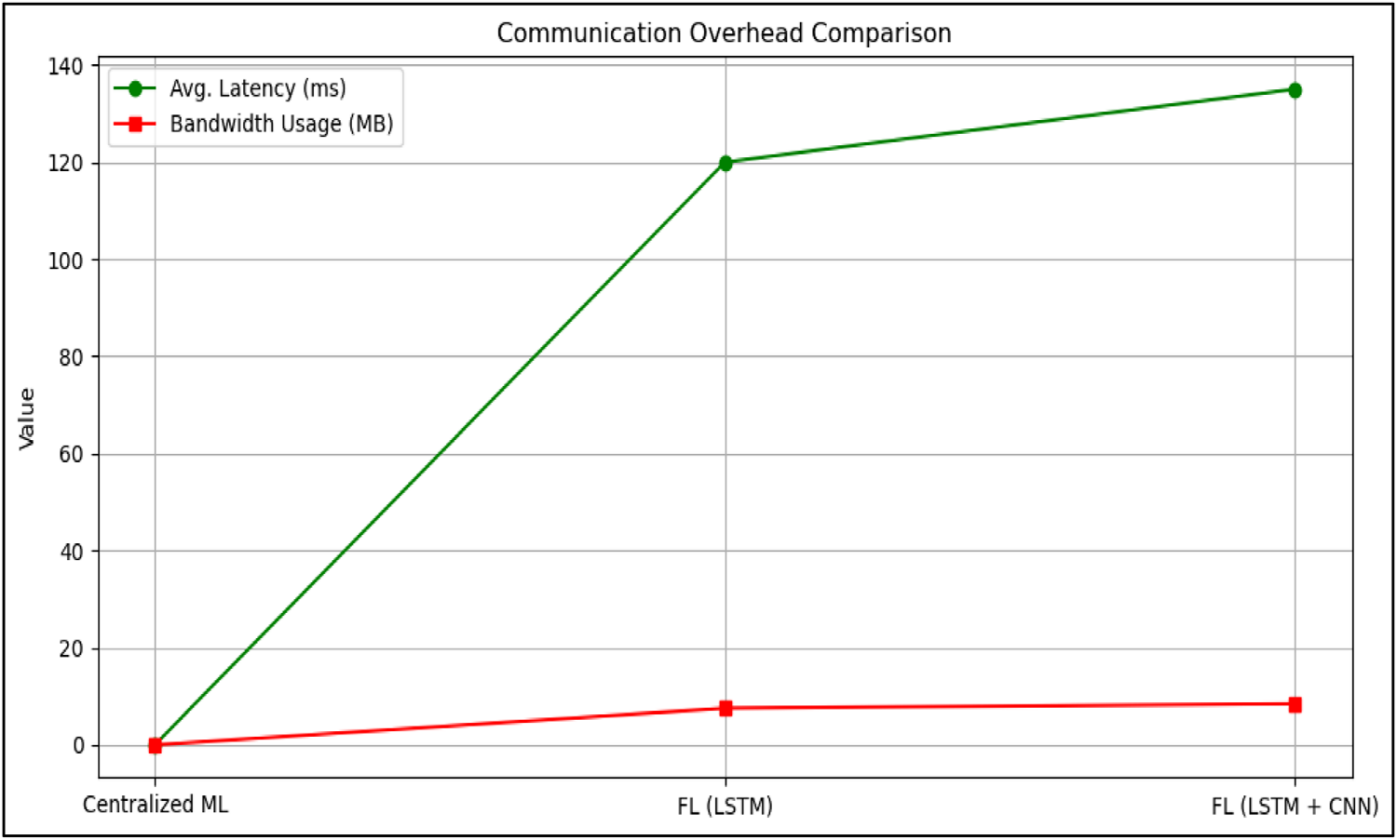
Latency and bandwidth comparison between centralized ML and FL models with LSTM and CNN.

**FIGURE 8:**
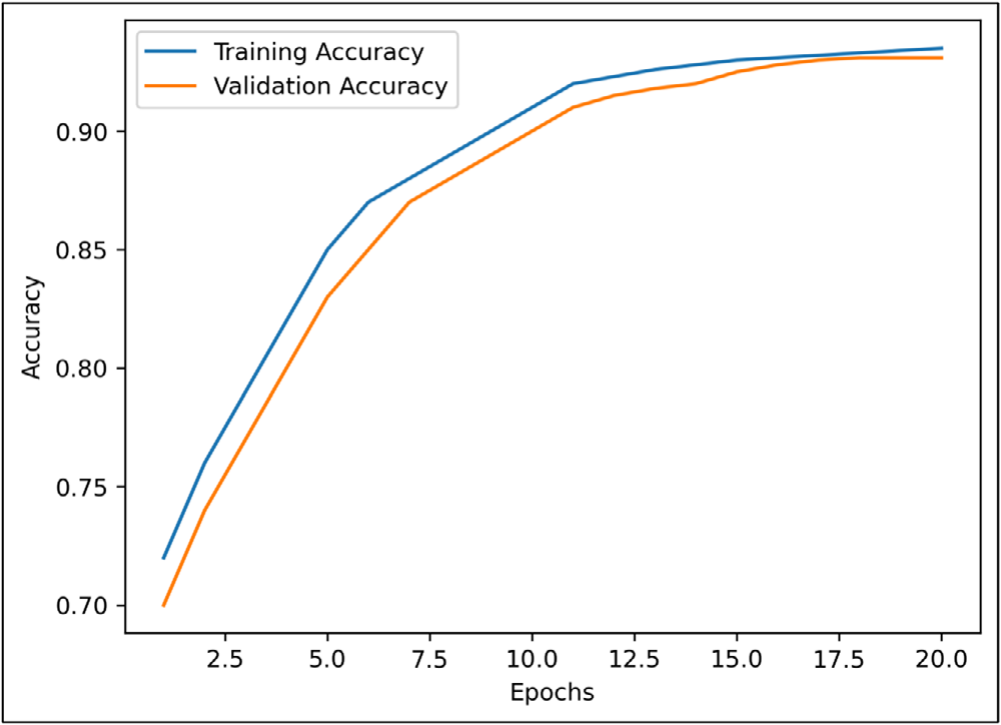
Training and validation accuracy of the proposed federated CNN–LSTM model on the MmCows dataset.

**FIGURE 9:**
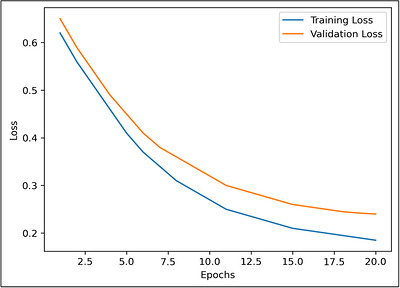
Training and validation loss curve of the proposed federated CNN–LSTM model on the MmCows dataset.

Figure 7 shows the latency and bandwidth usage comparison. Figures 8 and 9 shows training and validation accuracy and loss curve of the proposed Federated CNN–LSTM model on the MmCows dataset.

The proposed federated learning is mainly characterized by the size of model changes that are communicated between clients and the server, which determines the communication cost of the proposed architecture. The CNN–LSTM extensible model of a rough size of 1–2 MB, having 10 clients in the experiment and 50 communication rounds, overall uplink cost of communication is around 500–1000 MB with all the clients but the downlink cost is also of the same magnitude. These are economically viable on rural smart farming applications with disconnected connectivity particularly compared to the continuous transfer of raw data in centralized learning. In this context, communication efficiency can be defined as lowering the transmission of raw data via transmission of model parameters instead of the uninterrupted sensors, instead of lowering the absolute size of the model parameter transmission.

Although this work contrasts the proposed federated model with the representative traditional machine learning, centralized deep learning and generic federated learning baselines, more advanced federated optimization schemes (e.g., FedProx, SCAFFOLD, FedNova) and ablation studies were not done. This work is devoted to showing the relevance of federated learning to multimodal livestock monitoring in a limited privacy and connectivity setting. The comparison results in Table 12 show that deep learning models are superior to the ML because they have capacity to learn non‐linear and temporal features of multimodal sensor data which are difficult to learn by traditional ML methods. Our results demonstrate that the LSTM‐CNN hybrid FL model offers marginal advantages over the centralized model in terms of accuracy and F1‐score while maintaining data confidentiality and preventing data leakage. Although transmission delay and bandwidth consumption in FL exceed those in edge computing, they remain feasible for intermittent connectivity in smart farming applications.

**TABLE 12:** Comparison with traditional ML methods.

Model	Accuracy (%)	Precision	Recall	F1‐score
**SVM**	84.6	0.82	0.81	0.81
**Random forest**	87.9	0.85	0.86	0.85
**KNN**	82.3	0.80	0.78	0.79
**Centralized LSTM**	91.2	0.88	0.87	0.88
**FL (LSTM + CNN)**	93.1	0.92	0.90	0.91

In conclusion, our experiments show that federated learning, enhanced with powerful deep neural networks like LSTMs and CNNs, can perform high‐accuracy stress detection in cattle while improving privacy and maintaining reasonable system overhead. This architecture is well‐suited for practical smart livestock farming applications.

Although the performance of the proposed model is quite impressive, it should be pointed out that the stress labels involved in the present study are proxy labels based on physiological thresholds. Livestock stress is a complex process, which is not determined by physiological parameters alone (heart rate, body temperature, etc.), as well as by behavioural, hormonal and environmental indicators. Comprehensive stress evaluation of livestock, as it has been emphasized in the previous research, may frequently be carried out based on the combination of several physiological and welfare measures instead of relying on single threshold principles (Narayan et al. 2021).

### 6.1 Federated Training Configuration

To assure transparency and reproducibility of all experiments, the subsection Table 13 presents the federated learning configuration and the hyperparameter that was adopted in all experiments. In the proposed federated learning architecture, the clients are modelled as multi‐farm smart livestock systems, with each being a cattle and adjacent farmer‐gateway model, commonly referred to as an edge device, which locally trains the model on cattle‐specific sensor data.

**TABLE 13:** Federated learning configuration and hyperparameters.

Parameters	values
Number of federated clients	10 (simulated edge devices)
Client participation per round	100%
Communication rounds	50
Local training epochs	5
Batch size	32
Optimizer	Adam
Learning rate	0.001
Loss function	Binary cross‐entropy
Aggregation algorithm	FedAvg
Approx. model size	∼1–2 MB

The non‐IID non‐cattle‐identer partitioning of the MmCows data was done to reflect realistic heterogeneity across farms. Data on each of the federated clients belonged to a different set of animals, which led to client‐specific distributions of physiological, behavioural and environmental patterns. The design is a simulation of actual multi‐farm conditions in the real world where the properties of data differ owing to the animal behaviours, environment and management procedures.

### 6.2 Comparison of Proposed Work in Various Aspects

The comparison of the proposed work with previous studies along a number of pertinent aspects is summarized in the Table 14.

**TABLE 14:** Comparison of previous studies and proposed work in various aspects: technology used, data privacy, infrastructure dependency, data type, scalability and novelty.

Aspect	Study	Proposed work (FL‐based smart livestock monitoring)
**Technology used**	(Simanungkalit et al. 2021; Farooq et al. 2022; Post et al. 2020)—IoT with centralized ML models	Federated learning (FL) with multimodal data integration (This Study)
**Data privacy**	(Guitian et al. 2023; Wu et al. 2022; Bensakhria 2023)—Central data processing with privacy risks	Data stays on local devices; only model weights shared, ensuring privacy
**Infrastructure dependency**	(Unold et al. 2020; Khan et al. 2022)—Central/cloud‐based systems	Works in resource‐constrained environments with decentralized architecture
**Data types**	(Shahinfar et al. 2021; Guitian et al. 2023)—Single‐modal data (physiological or image)	Multimodal dataset (physiological, behavioural, environmental) from MmCows
**Stress detection methods**	(Acikmese and Alptekin 2019; Khan et al. 2022)—Used CNN/LSTM separately for stress/behaviour	Fusion of LSTM + CNN for unified stress detection (This Study)
**Real‐time capability**	(Post et al. 2020; Khan et al. 2022)– Cloud processing delays real‐time feedback	On‐device edge processing for real‐time health and stress monitoring
**Scalability**	(Bensakhria 2023; Arshad et al. 2023)—Centralized training limits scaling	Decentralized FL enables scalable learning across multiple farms
**Energy efficiency**	CowMonitor (Post et al. 2020)—Continuous communication to cloud is energy‐intensive	Optimized through on‐device inference and reduced data transmission
**Adaptability to farm environments**	(Mao et al. 2022; Guitian et al. 2023)—Poor suitability for rural or low‐connectivity areas	Designed for deployment in rural, bandwidth‐constrained environments
**Novelty**	(Dembani et al. 2025; Praharaj et al. 2025; Tangorra et al. 2024)—No FL with multimodal fusion for cattle	Multimodal fusion applied federated learning system to monitor cattle health and stress.

## 7 Conclusion and Future Work

Authors presented a FL technique for real‐time health and stress monitoring in cattle. The method processes multimodal time‐series and optional image/video data collected through IoT devices using a hybrid LSTM and CNN architecture in a federated environment. This approach addresses drawbacks of centralized ML models, particularly data privacy and issues related to limited internet connectivity in rural areas and communication efficiency.

Through extensive preprocessing, feature engineering and correlation analysis, we developed a resilient model capable of detecting high‐stress and abnormal behaviour in cattle. The FL model has attained similar accuracy and F1‐results compared to a centralized counterpart, without compromising raw data to the device level. This shows that FL can be a scalable and a secure solution to smart livestock farming.

Although this study is an experimental study to test the health and stress monitoring of cattle, future research will apply the proposed framework with empirical integration of the precision irrigation modules with soil moisture and environmental sensors. The system has real‐life applications that may be increased in the future. On‐site rapid inference could be done by deploying trained models on edge devices such as smart collars or micro controllers at the farm level. FL versions that are energy‐efficient, for example, FedProx, may reduce computation and communication overheads. The inclusion of other sensors (audio, thermal imaging, GPS) would help to present a more detailed explanation of the behaviour of cattle. Special prediction models might be used to enhance the quality of prediction on animals. Lastly, by incorporating this system into farm management systems and creating explainable AI tools that veterinarians can use, trust, usability and timely decision‐making would improve. Also, linking precision irrigation modules to the FL framework can ensure that water is used in the best way possible according to real time information on soil moisture, weather and the activities of livestock. In this way, farmers could manage both animals’ well‐being and their resources for better and sustainable farming.

## Author Contributions

**Vineeta Gulati**: conceptualization, formal analysis, methodology, project administration, software, visualization, writing – original draft. **Rahul Grover**: data curation, methodology, validation, writing – review and editing. **Naveen Kumar**: methodology, supervision, writing – review and editing. **Priya Jindal**: resources, supervision, writing – review and editing. **Momina Shaheen**: supervision, writing – review and editing.

## Funding

The authors have nothing to report.

## Ethics Statement

The authors have nothing to report.

## Conflicts of Interest

The authors declare no conflicts of interest.

## Data Availability

The data that support the findings of this study are openly available in Zenodo at https://doi.org/10.5281/zenodo.15679892, reference number (Polsky and Von Keyserlingk 2017).
